# Design and Experimentation with Sandwich Microstructure for Catalytic Combustion-Type Gas Sensors

**DOI:** 10.3390/s140305183

**Published:** 2014-03-12

**Authors:** Jun-Tao Gu, Yong-De Zhang, Jin-Gang Jiang

**Affiliations:** 1 Intelligent Machine Institute, Harbin University of Science and Technology, Xuefu Road No. 52, Nangang District, Harbin 150080, China; E-Mails: gujt9@126.com (J.-T.G.); zhangyd@hrbust.edu.cn (Y.-D.Z.); 2 Heilongjiang Province Electronic & Information Products Supervision Inspection Institute, Harbin 150090, China

**Keywords:** sandwich structure, micro double bridge, catalytic combustion, gas sensor

## Abstract

The traditional handmade catalytic combustion gas sensor has some problems such as a pairing difficulty, poor consistency, high power consumption, and not being interchangeable. To address these issues, integrated double catalytic combustion of alcohol gas sensor was designed and manufactured using silicon micro-electro-mechanical systems (MEMS) technology. The temperature field of the sensor is analyzed using the ANSYS finite element analysis method. In this work, the silicon oxide-PECVD-oxidation technique is used to manufacture a SiO_2_-Si_3_N_2_-SiO_2_ microstructure carrier with a sandwich structure, while wet etching silicon is used to form a beam structure to reduce the heat consumption. Thin-film technology is adopted to manufacture the platinum-film sensitive resistance. Nano Al_2_O_3_-ZrO-ThO is coated to format the sensor carrier, and the sensitive unit is dipped in a Pt-Pd catalyst solution to form the catalytic sensitive bridge arm. Meanwhile the uncoated catalyst carrier is considered as the reference unit, realizing an integrated chip based on a micro double bridge and forming sensors. The lines of the Pt thin-film resistance have been observed with an electronic microscope. The compensation of the sensitive material carriers and compensation materials have been analyzed using an energy spectrum. The results show that the alcohol sensor can detect a volume fraction between 0 and 4,500 × 10^−6^ and has good linear output characteristic. The temperature ranges from −20 to +40 °C. The humidity ranges from 30% to 85% RH. The zero output of the sensor is less than ±2.0% FS. The power consumption is ≤0.2 W, and both the response and recovery time are approximately 20 s.

## 1. Introduction

The sensor is the front-end and key component to acquire information in modern information systems, and its performance is directly related to the quality of the information system's perception of external data [1]. Therefore, the design and manufacture of the sensor are key to any information technology research. In addition, the design of gas sensors is particularly difficult because the sensitive body must come into contact with the atmospheric environment [2].

For the detection of a certain gas, we can use either of the photoelectric principles, *i.e.*, the electrochemical or catalytic combustion method, to design the semiconductor [3]. In contrast, for combustible gas, the catalytic combustion sensor has a higher sensitivity and faster recovery response when compared with others. Furthermore, the manufacturing technology is simple and inexpensive, which results in the largest output and a wider range of applications.

The traditional catalytic combustion gas sensor is comprised of a metal oxide catalyst carrier material, precious metal catalyst, and sensitive elements that offer heat sensitive resistance. When using the sensor, its carrier requires a reference cell comprising another identical resistance, which is insensitive to flammable gases, to constitute a paired device [4]. The signal difference in the two units is compared through a circuit amplifier to obtain the relationship between the electronic signal and the gas concentration as well as to perform the detection [5].

The traditional design of the catalytic combustion gas sensors is carried out by coating the carrier and catalyst materials on the heated platinum wire by hand. Afterwards, a heat treatment process cures the composition sensitive elements. This will serve as the reference unit. Meanwhile, the heated platinum wire coil is only coated with a carrier material that is not sensitive to flammable gases [6]. The traditional gas sensors are handmade, therefore such traditional gas sensors exhibit pairing difficulties, poor consistency, and high power consumption problems. There is no solution yet to the interchangeability problem [7].

Since the 1990s, with the development and maturation of silicon MEMS technology, silicon micromachining technology entered the micromachining field. The devices have the characteristics of miniaturization, integration, low power consumption, and low mass [8]. Researchers began attempting to manufacture gas sensors using the silicon MEMS sensor technology, and these gas sensors were applied in systems for sensor microstructures [9]. Manginell, who worked at Sandia National Laboratory, came up with the concept of adopting the microbridge fine silk method to manufacture a catalytic burning-type hydrogen sensor [10,11]. The experiment was based on micro-processing technology, according to the shape of micro-bridge fine silk graphics carved micro-bridge fine silk. The catalyst coated used platinum acetyl acetone steam selective chemical gas phase deposition. The coated catalyst was able to produce 70 mW of micro-power for the catalytic type hydrogen gas sensor.

In this work, single-crystal silicon is used to produce sensors with a SiO_2_/Si_3_N_4_/SiO_2_ sandwich structure, which reduces the structural influence due to the high temperature. Through coating and heat processing technology, the carrier material is formed, and the catalyst on the chip is cured, thereby producing a new type of microbridge structure for catalytic combustion gas sensors. The data from the changing output voltage of the sensor owing to the gas concentration are acquired through circuits, and the gas sensor sensitivity to alcohol vapor is analyzed.

## 2. Experimental Methods

### 2.1. Working Mechanism Analysis

This catalytic combustion gas sensor comprises of sensitive and reference cells that are integrated on a chip, and it is composed of two isolation bridge beams. Each of the beams of the bridge has a platinum thin-film thermal resistor with the same resistance value.

The platinum thin-film thermal resistor on the beam of the bridge is heated through an external circuit, in order for it to reach a constant temperature range between 300 and 350 °C. At this time, according to Equation (1) the platinum resistance corresponds to the following resistor value

(1) $$R_{t}=R_{0}\left(1+\alpha t+\beta t^{2}\right)$$

The resistance value is *R_t_* when the temperature is *t*, *R*_0_ is the nominal resistance when the temperature is zero, and α is the temperature coefficient when the platinum sensitive resistance is being heated. β is double the value of the resistance temperature coefficient, and γ is triple the value of the resistance temperature coefficient.

The sensitive and reference elements have the same platinum thin-film thermal resistor value; therefore, the difference in the resistance values of the two elements is always zero at any constant temperature. However, when the sensor is exposed to flammable gases such as ethanol vapor the reference unit becomes insensitive to it. This makes the platinum thin-film thermal resistor for the beam of the bridge remain constant. The sensitive elements play the role of the catalyst. The oxidation reaction of the combustible gases such as alcohol with oxygen in the air occurs in the sensitive cell surface. The catalytic combustion reaction equation is

(2) $${2\text{C}}_{\text{x}}{\text{H}}_{\text{y}}+(2\text{x}+\frac{\text{y}}{2}){\text{O}}_{2}\frac{\text{catalyst}}{\Delta }{2\text{xCO}}_{2}{+\text{yH}}_{2}\text{O}+\text{Q}$$

The oxidation reaction produces heat (flameless catalytic thermal combustion), which increases the resistance of the platinum thin-film thermal resistor as the temperature increases. Thus, there exists a difference in the resistances of the sensitive and reference elements. When there is no flammable gas; the resistance difference becomes zero, and the ratio of the resistance difference and combustible gas concentration is one to one.

Generally, the catalytic combustion gas sensor adopts the Wheatstone bridge method to collect the signal [12,13], which keeps track of the sensitive cells with the platinum thin-film thermal resistor changes owing to the changes in the oxidation reaction producing heat, which reflects the essential features. However, the temperature of the sensor has a long time delay; therefore, the resistance changes leads to a long signal response time. Furthermore, the catalyst carrier material on the platinum thin film and catalyst can also make the surface become unstable carbon owing to the frequent changes in temperature, thereby reducing the sensor performance.

**Figure 1.**
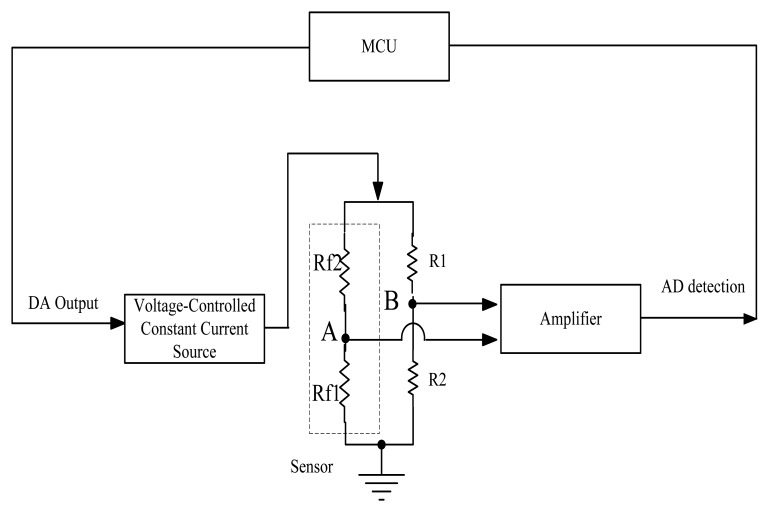
Temperature detection circuit diagram for sensor signal acquisition.

On the basis of the preceding analysis, in this work, a temperature detection circuit for the sensor signal acquisition has been designed, as shown in Figure 1.

Rf1 is the sensitive element resistance shown in Figure 1, Rf2 is the reference element resistance, and R1 and R2 are the precision resistors of the same value of the balance bridge. The gas induces an exothermic oxidation reaction on the catalytic sensing element surface, which causes the catalytic sensor temperature to rise, the resistance value rise, and the output of the bridge to be unbalanced. When the AD detects a change in the operational amplifier, it will change the output voltage of the DA. Thus, the current output of the pressure-controlled constant current source reduces its temperature and resistance value, until the bridge recovers to a balanced resistance. Through this feedback, the catalytic sensors work at a constant temperature.

When the sensitive resistance Rf1 comes into contact with the alcohol gas, the temperature of the sensitive elements and resistance value rises because of the abrupt oxidation (burning) releasing heat. Therefore, the bridge circuits are no longer balanced. Because of this, the bridge circuits produce an electric potential *E* between A and B, which is expressed as

(3) $$E=k(\frac{R_{F_{2}}}{R_{F1}})\Delta R_{F}$$

The electric potential between A and B is approximately proportional toΔ*R_F_*. Δ*R_F_* emerges owing to the changes in the temperature because of the catalytic fuel gas combustion (combustion heat), which is proportional to the catalytic combustion heat (flammable gases emit carbon dioxide). Δ*R_F_* can be expressed as

(4) $$\Delta R_{F}=\alpha \Delta T=\alpha \frac{\Delta H}{C}=\alpha am(\frac{Q}{C})$$

where α is the resistance temperature coefficient of the sensitive element, Δ*T* is the value of the temperature increase owing to the alcohol gas catalytic combustion, Δ*H* is the heat produced from the alcohol gas catalytic combustion, *C* is the heat capacity of the sensitive elements, *Q* is the heat of combustion of the alcohol gas, *M* is the concentration (volume fraction) of the alcohol gas, and a is a constant, which depends on the catalyst coated on the sensitive cells.

*C* and *a* are related to the material, shape, structure, surface treatment, and other factors of the sensitive elements because of the value of *α*. Meanwhile, *Q* is determined by the type of alcohol gas. In certain circumstances, *K* = *αa*(Q/C) is a constant; therefore,

(5) $$E=\beta \Delta R_{F}=\beta Kq$$

where *β* is the current ratio of the sensitive elements The potential difference between the points A and B is proportional to the concentrations m of alcohol vapor. When a voltmeter is connected between the points A and B, we can measure the E between points A and B and obtain the alcohol vapor concentration in the air, and through a standard correction, we can accurately detect the concentration of the alcohol vapor.

### 2.2. Structural Design and Simulation of the Sensor

**Figure 2.**
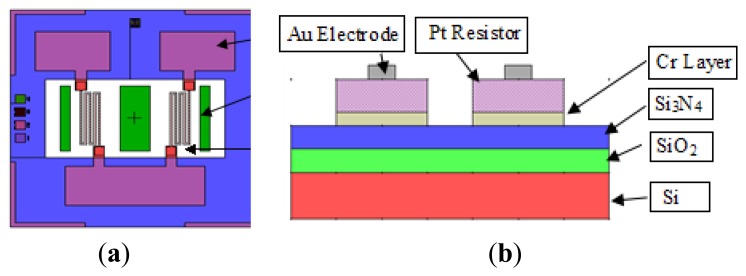
Chip domain of the micro double bridge catalyzing the alcohol gas. (**a**) Configuration; (**b**) Side view.

The power consumption of the traditional catalytic combustion sensors is greater than 0.7 W, and the temperature and humidity interference degree is approximately 5%. Therefore, for the structural design of the sensors, we have considered the low power requirements of the catalytic combustion-type gas sensor, and reduced the heated sensor chip power effectively by reducing the formation of thin insulated tanks, and reduced the metal electrode width to reduce the cooling efficiency. The sensor comprises of a sensitive leg with a catalyst and a catalyst leg without compensation. The bridge arm size is 0.2 mm × 0.5 mm, and its thickness is 30 μm (containing 20 μg/m of single-crystal silicon). Figure 2 shows the integrated dual catalytic alcohol-sensing chip layout design. The chip comprises of a silica leg, heat-sensitive electrode and catalyst carriers, and catalysts, at the front. There are a total of two signal electrode leads at the front of the chip.

The catalytic combustion-type gas sensor is a device that works in hot environment, and the chip has a heat sensitive resistance. With the power on, it forms a temperature field between 300 and 350 °C. Therefore, in the structural design, it is necessary to consider the heating power and temperature relations. Simultaneously, we must consider the influence of the material properties, materials, size, thickness of the electrode, vector of the heat distribution, and structural stress and strain. Under ideal conditions, the chip sensitive units and unit of temperature are limited by the local area; therefore, they do not affect each other, and the heat does not reach the base zone.

In this work, the ANSYS finite element method is used to analyze the sensor temperature. The simulation parameters are determined as follows: Pt film thick = 1 μm; Au electrode film thick = 3–5 μm; nanolevel chrome layer, nitride silicon thickness = 1 μm, SiO_2_ thickness = 2 μm, Si thickness = 96 μm (reduced thin zone), 500 μm of non-reduced thin zone; Al_2_O_3_ powder body slurry thick is approximately 0.2 mm, drops painted in Pt resistance article district; Au silk lead connection package shell Shang made of copper wire, Au silk diameter = 0.06 mm; environmental temperature is 27 °C, and the remaining size is based on the map size.

**Figure 3.**
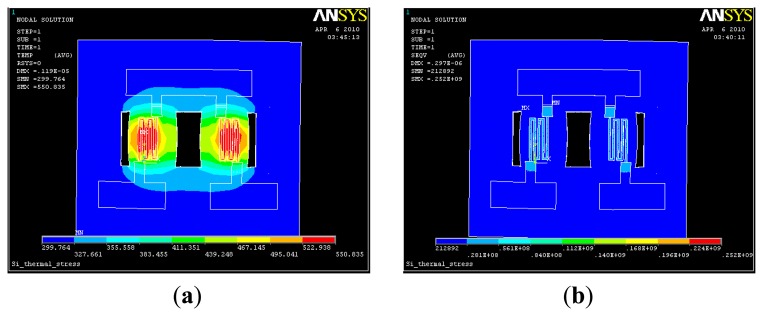
Sensor temperature distribution and the stress distribution diagram. (**a**) Temperature profile; (**b**) Stress envelope.

A sensor model is established, and a 1.5-V heating voltage is applied to the sensor. Further, we have calculated the heating resistor R to be approximately 12 Ω, the power consumption is approximately 0.19 W, and the surface temperature is 350 °C. The temperature distribution images and stress distribution of the clouds are shown in Figure 3. The heat is focused on the leg, the temperature on the two bridges is uniform relatively, and the stress is mainly distributed in the platinum resistance electrode and between the layers.

**Figure 4.**
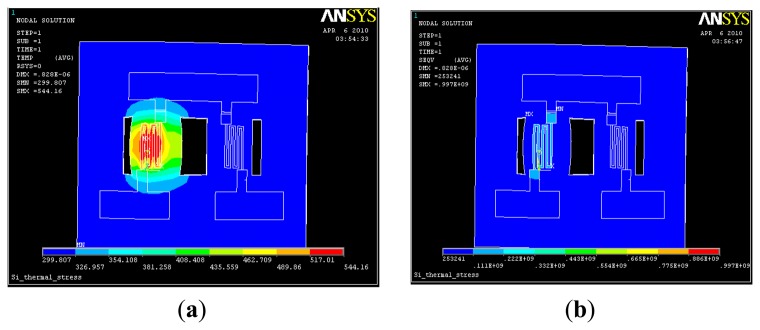
Analysis diagram of the thermal interference between the two bridges of the sensor. (**a**) Temperature profile; (**b**) Stress envelope.

When the heating voltage is only applied to one of the bridges, simulation of the temperature and stress distribution in the structure can aid in the analysis of the thermal interference between the two bridges. The simulation results are shown in Figure 4.

A single cantilever bridge is heated at a lower temperature, *i.e.*, approximately 60 °C, compared with the simultaneous heating of two cantilever bridges. Therefore, it can be observed that the double-bridge has a small thermal interference; however, for the platinum film thermal resistance, it is possible to tell the difference. Therefore, acquiring the information through a constant temperature detection circuit is necessary.

### 2.3. Sensor Manufacture Process

Catalytic combustion-type gas sensor manufacture includes microprocessing chips, platinum film electrodes, and synthetic sensitive material.

#### 2.3.1. Chip Microstructure Manufacture

**Figure 5.**
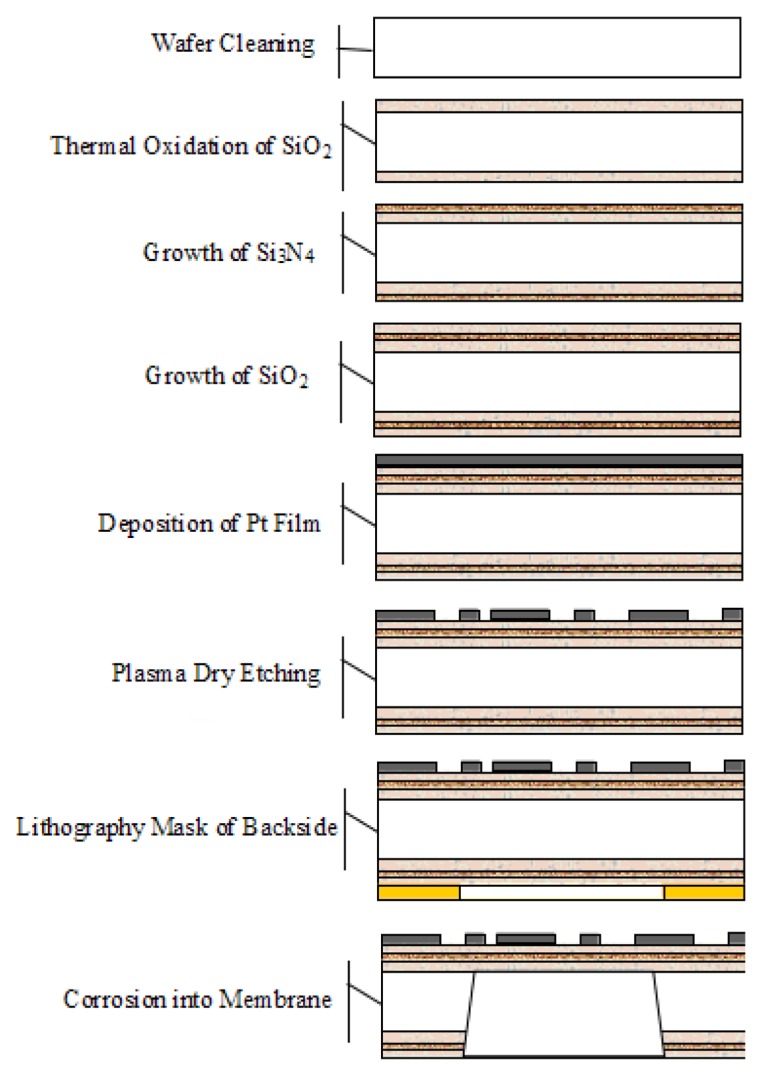
Flow-process diagram of creating a chip.

The microprocessing chips are related to the photolithography and etching process. First, a layer of SiO_2_ is oxidized, and then Si_3_N_4_/SiO_2_ is deposited, which forms the sandwich structure. This process is shown in Figure 5.

The manufacturing process integrates the semiconductor silicon micromachining technology and thin-film technology to form the bridge advances. This is accomplished by using the wet method to create a 5-μm oxidation layer, PECVD to create a 3-μm layer of SiO_2_ oxidation, and lithography flooded wet etching to create the SiO_2_/Si_3_N_4_/SiO_2_ sandwich structure.

Through electron beam evaporation, pure platinum, whose thickness is approximately 0.7 to 0.8 μm, is evaporated through photoresist masks, plasma etching, and heat treatment, thereby forming a thin-film thermal resistor. Laser etching technology is used to adjust the thermistor from −0 to 10∼11 Ω. Ion beam etching forms the thermal resistor. Lithography masks the backside using the wet method etching of the SiO_2_/Si_3_N_4_/SiO_2_ sandwich, Then, the semiconductor monocrystalline silicon is etched with KOH solution. The film is released to form a hanging bridge, which forms a usable microbridge structure.

#### 2.3.2. Sensitive Material Synthesis and Catalyst Coating

Although the carrier is not directly involved in the catalytic combustion reaction, it plays the role of supporting the coating and catalyst; therefore, it provides a large surface and space for the three-dimensional spatial distribution of the catalyst. Thus, the type of carrier material directly affects the activity of the catalyst. γ-Al_2_O_3_ is a good industrial catalyst carrier because it has excellent properties, good high temperature performance, and a large surface area. We can mix Mg^2+^, Ce^2+^, Sb^3+^, and Zr^2+^ plasma to improve its catalytic function and activity.

In this work, sol-gel fabrication is used to produce Al_2_O_3_, which is a type of ultrafine functional powder, and produce Al(OH)_3_ precipitate colloidal base on Al_2_(SO_4_)_3_. Through the heat process, Al_2_(SO_4_)_3_ becomes γ-Al_2_O_3_ nanopowder. The reaction equations are:

(6) $${\text{Al}}_{2}{{(\text{SO}}_{4})}_{3}+{\text{NH}}_{3}\cdot {\text{H}}_{2}\text{O}\overset{\text{catalyst}}{\to }{\text{Al}(\text{OH})}_{3}+{\left({\text{NH}}_{4}\right)}_{2}{\text{SO}}_{4}$$

(7) $$\text{Al}{\left(\text{OH}\right)}_{3}\overset{\Delta }{\to }{\text{Al}}_{2}{\text{O}}_{3}{+\text{H}}_{2}\text{O}$$

γ-Al_2_O_3_ is mixed with ZrO_2_. ThO is mobilized using a specific fixture that is evenly coated on the microdouble thermistor. Then, the thermistor is sintered by passing a 3.5∼4 V voltage through the thermistor. Afterwards, a 1% Moore concentration of chloroplatinic acid is prepared. Then, little drops of it are added to the carrier film of the sensitive bridge arm. Another 2% Moore concentration of lead acetate is prepared, and little drops of it are added in the carrier film of another bridge arm, which forms a compensation unit. After drying in the shade, a 3.5∼4 V voltage is passed through the thermistor for 10 to 20 min.

#### 2.3.3. Sensor Chip Package

**Figure 6.**
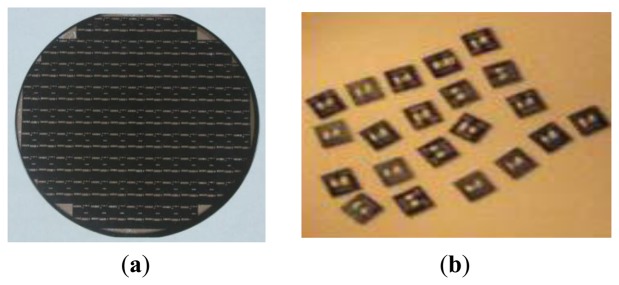
The microstructure of the double-bridge sensor. (**a**) 4-in silicon chips; (**b**) Separated chips.

The sensor chip was silicon base and a 4-in wafer. Using the wheel scribing process, the chip is divided into small structures. We can improve the stability of the film electrode using a high-temperature processing atmosphere with an annealing temperature of 300∼350 °C, and an annealing time of 2 h. Figure 6 shows the sensor microstructure with a microbridge structure comprising a 4-in silicon chip. The size of the chip is 5 mm × 5 mm, and the effective microbridge size of the sensitive elements and reference unit is 0.5 × 1.2 × 0.08 (mm).

**Figure 7.**
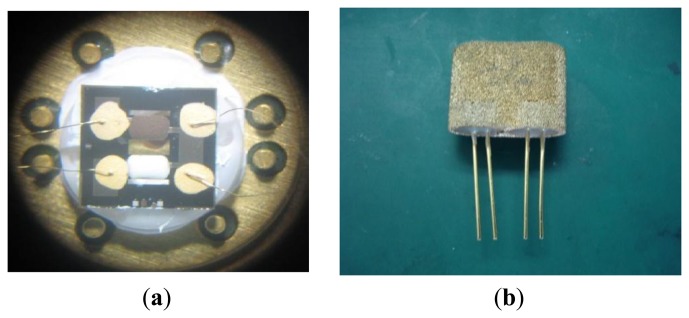
Photograph of the transducer. (**a**) Before packaging; (**b**) After packaging.

The common solder process cannot guarantee reliability due to the 300∼350 °C operating temperature of the chip. Therefore, we adopt a relatively simple method to combine gold wire welding with Au plasma welding. A 0.06-mm gold wire is used as the lead. The welding temperature is 500 °C, while sintering lasts 20 min. Then, the capacitor discharge technology is used to weld the chip lead in the sensor base and package. Figure 7 shows a picture of the sensor.

## 3. Results and Discussion

### 3.1. Analysis of the Surface Morphology and Composition

In general, using chemical corrosion to corrode the Pt thin-film resistance is difficult. In addition, the main issue with the chemical corrosion process is that the corrosive liquid has to synchronize the injury to the photoresist layer. However, the general-purpose test methods cannot be used to the rule map.

**Figure 8.**
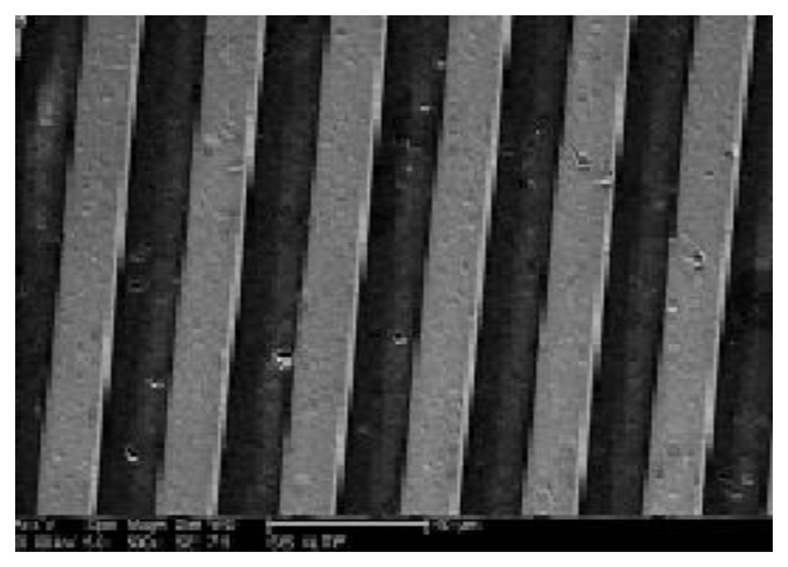
Micrographs of the Pt thin-film resistor lines.

In this work, the physical dry-etching process routing is used along with ion beam etching to solve the Pt film layout problems. Figure 8 shows a Pt thin-film resistor for ion beam etching and microscopic photos of the line. We can observe that the etching width is within 2 μm, contains structured lines, and did not harm the platinum film crystals, thereby guaranteeing the clarity, completeness, and consistency of the platinum resistance layout.

**Figure 9.**
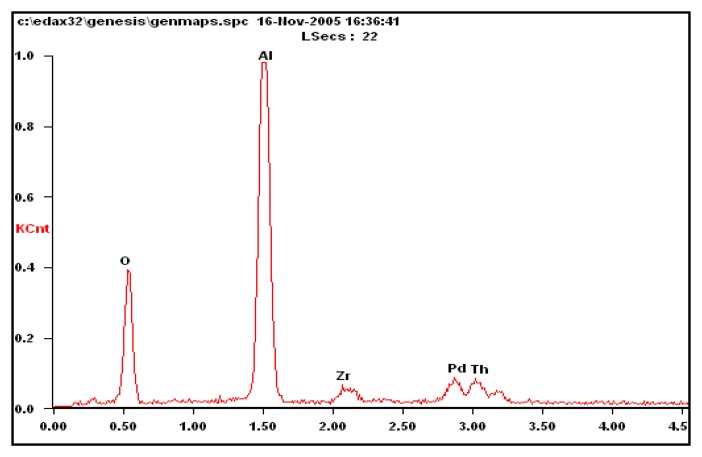
Scanning electron microscope energy spectrum analysis results from the carrier of the sensitive bridge arm.

**Figure 10.**
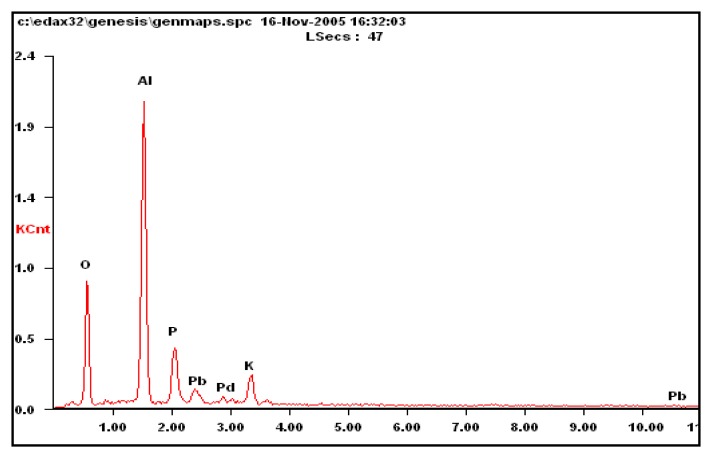
Scanning electron microscope energy spectrum analysis results from the carrier of the compensation bridge arm.

The power mirror energy spectrum is used to analyze the composition of the carrier sensitive materials and carrier compensation material. The results are shown in Figures 9 and 10. The composition of the carrier and catalyst on the sensitive bridge arm is Al_2_O_3_-ZrO-ThO-Pd. The composition of the carrier and wet to min-agent on the compensation bridge arm is Al_2_O_3_-PbO-Pd.

### 3.2. Signal Acquisition Circuit

**Figure 11.**
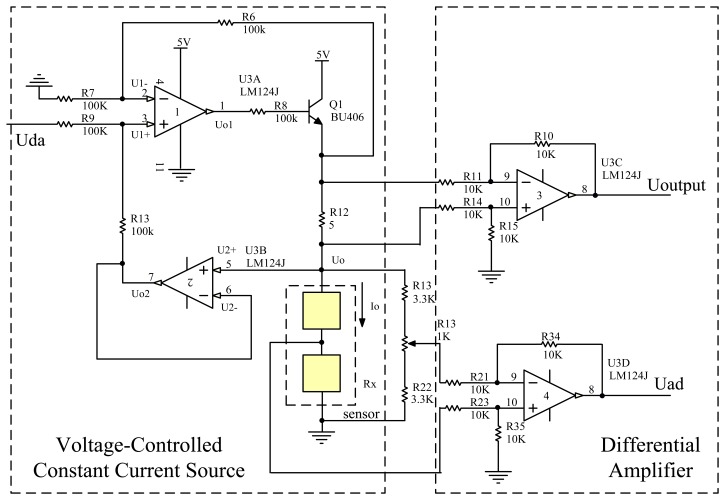
Sensor signal acquisition circuit.

**Figure 12.**
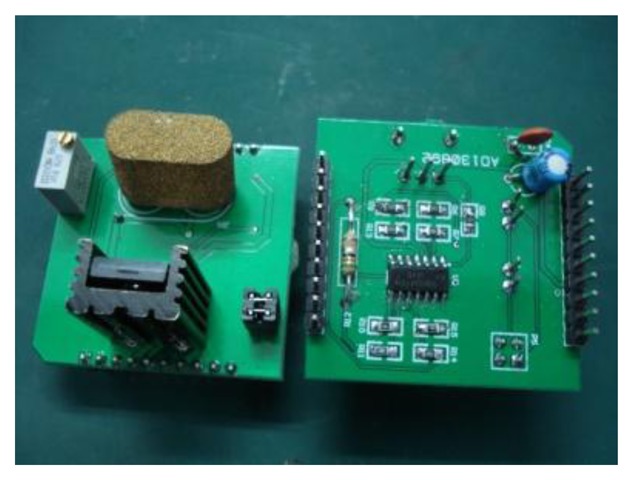
Actual photos of the circuits.

The output signal ranges from 0 to 80 mV because the sensor works in a specific range; therefore, it is necessary to acquire, amplify, and compensate the signal. The circuit designed has to measure a temperature range of −50 °C to 100 °C. Figure 11 shows the signal collection circuit, and the overall circuit includes a voltage-controlled constant current circuit and differential amplifier circuit. Figure 12 shows an actual photos of the circuits.

The voltage-controlled constant current circuit includes an LM124 operational amplifier and a BU406 transistor, which allows for a maximum current output of 250 mA. The differential amplifier circuit is used to amplify the sampling resistance as well as the bridge output. U3B is a follower; therefore,

(8) $$U_{2+}=U_{o2}$$

By taking advantage of the virtual break concepts of the operational amplifiers, we can know that

(9) $$\frac{U_{DA}-U_{1+}}{R_{9}}=\frac{U_{1+}-U_{o2}}{R_{13}}$$

(10) $$\frac{U_{1-}}{R_{7}}=\frac{U_{o1}-U_{1-}}{R_{6}}$$

By taking advantage of the virtual brief concepts of the operational amplifiers, we can know that

(11) $$U_{1+}=U_{1-}$$

Because *R*_6_ = *R*_7_ = *R*_9_ = *R*_13_ and solving Equations (9) and (10), we can know that

(12) $$U_{1+}=\frac{U_{DA}+U_{o2}}{2}$$

(13) $$U_{1-}=\frac{U_{o1}}{2}$$

Because $U_{1+}=U_{1-}$, $\frac{U_{DA}+U_{o2}}{2}=\frac{U_{o1}}{2}$ and $U_{o1}-U_{o2}=U_{DA}$. Thus, there is a relationship between the two voltage points of *R*_12_, *i.e.*, $U_{o1}-U_{o}=U_{o1}-U_{o2}=U_{\text{DA}}$.

By taking advantage of the virtual break concepts of the operational amplifiers, we can know that the current passing through *R*_12_ is equal to the current passing through the sensor, *i.e.*, $I_{o}=\frac{U_{o1}-U_{o}}{R_{12}}=\frac{U_{DA}}{R_{x}}$. When *R*_12_ is a constant, we can change the output of the constant current source by changing the output voltage of the DA. In this manner, we can change the current that passes through the sensor. BU406 is used to amplify the current, which is the sampling resistance.

The differential amplifier output can be expressed as

(14) $$U_{\text{output}}=\frac{R_{10}}{R_{11}}(U_{o1}-U_{o})$$

By amplifying the voltage of the sampling resistance, we can guarantee that the output voltage ranges from 1 to 3 V.

### 3.3. Performance Test Results

A specific dynamic test system for gas sensors is used in conjunction with the standard gas dilution device to produce a volume fraction (0∼10,000) of X10-6 standard ethanol gas. The gas chromatographic detection of the final alcohol concentrations is observed.

**Figure 13.**
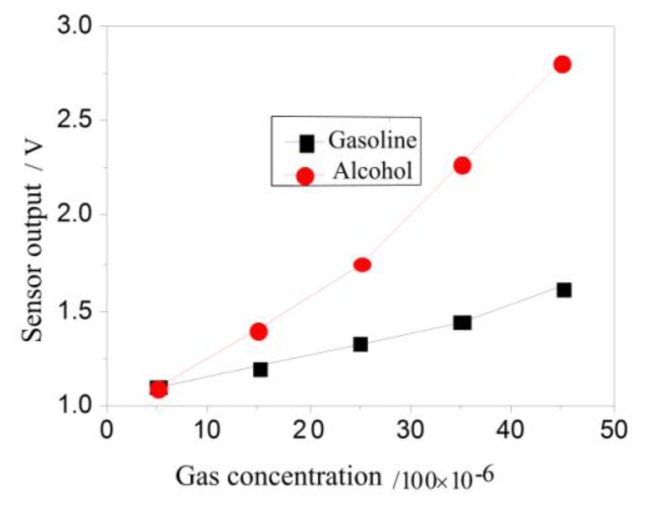
Relationship between the sensor output and the gas volume fraction.

Figure 13 shows the corresponding alcohol and gasoline outputs when the sensors for a 1.5-V operating voltage, 25 ± 1 °C environmental temperature, 30% ± 3% RH humidity, and 150-mL/min gas flow. From the characteristic curve, we can observe that the sensor's output is linear when the concentrations of the alcohol and petrol are equal. The voltage output change rate of alcohol is significantly higher than that of gasoline, and the former is more sensitive and more selective than the latter.

**Figure 14.**
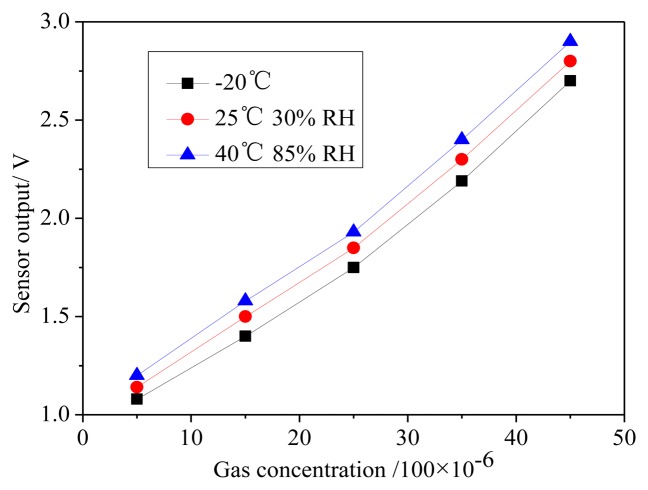
Relationship between the sensor output, temperature, and humidity.

We used an ESPEC constant temperature humidity chamber to test the alcohol sensor for the humidity and temperature characteristics. We set the humidity uncertainty to ±3%, temperature uncertainty to 1.0 °C, at 40∼85 RH% (high temperature and high humidity), 25∼30 RH% (common temperature and common humidity), and −20 °C (low temperature environment), to test the alcohol sensor output. The sensor output comparison data are as shown in Figure 14. From the results, we can observe that the maximum output volatility produced by the interference originates from the changes in the environment condition, which is less than 0.35 mV or equivalent to 2% on the full scale.

**Figure 15.**
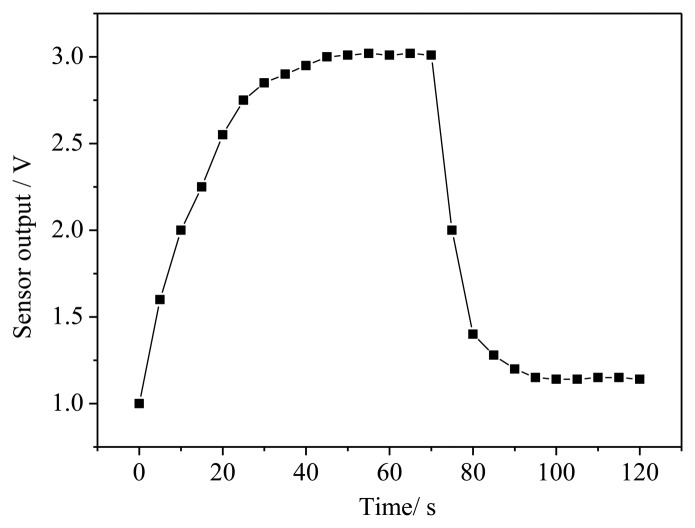
Sensor response curve vs. recovery time.

The alcohol sensor response time-to-recovery test uses a dedicated dynamic test system. The sensor output accesses the data capture card of the PC and the gas density using a 4,500 × 10^−6^ volume fraction. The results are shown in Figure 15. From the test results, we can observe that when the alcohol sensor experiences a 2.5 V output, the recovery time is approximately 20 s.

## 4. Conclusions

Through the use of the silicon micromachining technology, an integrated microbridge structure is formed. Plane sputtering and etching technology are used to improve the bridge arm thermistor. A constant-temperature bridge signal processing circuit was manufactured. The sensor has many advantages such as a linear output and good selectivity to alcohol vapor. When compared with the traditional handmade sensor, the interference temperature and humidity are reduced to 2%, the response time is less than 30 s, and the power is within 0.2 W. The sensor has stable operation and good consistency. This provides the technical support for production and puts forth a new method for the design of a combustible gas sensor.
